# A major-capsid-protein-based multiplex PCR assay for rapid identification of selected virulent bacteriophage types

**DOI:** 10.1007/s00705-019-04148-6

**Published:** 2019-01-23

**Authors:** Yannick Born, Leandra E. Knecht, Mirjam Eigenmann, Michel Bolliger, Jochen Klumpp, Lars Fieseler

**Affiliations:** 1 Institute of Food and Beverage Innovation, Zurich University of Applied Sciences, 8820 Wädenswil, Switzerland; 2 Institute of Food, Nutrition and Health, ETH Zurich, Zurich, Switzerland

## Abstract

Bacteriophages represent a promising alternative for controlling pathogenic bacteria. They are ubiquitous in the environment, and their isolation is usually simple and fast. However, not every phage is suitable for biocontrol applications. It must be virulent (i.e., strictly lytic), non-transducing, and safe. We have developed a method for identifying selected types of virulent phages at an early stage of the isolation process to simplify the search for suitable candidates. Using the major capsid protein (MCP) as a phylogenetic marker, we designed degenerate primers for the identification of Felix O1-, GJ1-, N4-, SP6-, T4-, T7-, and Vi1-like phages in multiplex PCR setups with single phage plaques as templates. Performance of the MCP PCR assay was evaluated with a set of 26 well-characterized phages. Neither false-positive nor false-negative results were obtained. In addition, 154 phages from enrichment cultures from various environmental samples were subjected to MCP PCR analysis. Eight of them, specific for *Salmonella enterica*, *Escherichia coli*, or *Erwinia amylovora*, belonged to one of the selected phage types. Their PCR-based identification was successfully confirmed by pulsed-field gel electrophoresis of the phage genomes, electron microscopy, and sequencing of the amplified *mcp* gene fragment. The MCP PCR assay was shown to be a simple method for preliminary assignment of new phages to a certain group and thus to identify candidates for biocontrol immediately after their isolation. Given that sufficient sequence data are available, this method can be extended to any phage group of interest.

**Electronic supplementary material:**

The online version of this article (10.1007/s00705-019-04148-6) contains supplementary material, which is available to authorized users.

## Introduction

Bacteriophages (phages) are viruses that infect only bacteria. Due to the rise of antibiotic resistance in many relevant bacterial pathogens, interest in phage research is steadily increasing. Because of their unique properties, phages are promising alternatives for the control of bacteria. The major advantage is their host specificity, which enables a targeted treatment of a pathogen. In addition, phages are self-limiting and natural [1]. However, not every phage is suited for application as a biocontrol agent. In general, biocontrol phages need to be virulent (i.e., strictly lytic) and non-transducing, and they should not carry genes known to encode toxins. They also must be stable during storage and application. Finally, they need to be propagatable to high titers on non-pathogenic production strains [2]. Phage biocontrol has been successfully applied in food pre- and postharvest to control the major foodborne pathogens *Salmonella enterica*, shiga-toxin-producing *E. coli* (STEC), *Campylobacter jejuni*, *Listeria monocytogenes,* and *Staphylococcus aureus* [2]. Likewise, phages have been used to control plant infections caused by *Xanthomonas* spp. or *Erwinia amylovora* [3], and they have been applied in aquaculture to treat bacterial infections of fish and shellfish [4].

The total number of phages on Earth is enormous and exceeds an estimated 10^31^ virions [5, 6]. Given this vast number, it is likely that there is a phage specific for any host that meets all the demands on a biocontrol phage. In general, phages can be easily isolated from environmental samples. Protocols for isolation of tailed phages (members of the order *Caudovirales*) typically involve an enrichment step followed by plating using the soft-agar overlay method [7, 8]. Standard enrichments are usually performed with a single bacterial strain, while protocols adjusted towards isolation of polyvalent phages include sequential enrichment with several host strains [7, 9]. Both methods normally yield numerous isolates. However, the subsequent characterization of all isolates to avoid redundancy and to identify virulent candidates is time consuming and laborious.

The increase in the number of completely sequenced phage genomes in the past years has enabled comparisons of whole genome sequences, which has revealed evolutionary relationships among different phage groups. Although clearer parameters to define genera, subfamilies, and families still need to be established, new phage subfamilies and genera have been proposed on the basis of molecular relationships (e.g., phages with 40% homologous proteins are members of the same genus) [10]. Importantly, members of the same subfamily or genus usually share a lifestyle and approximate genome size [11]. For example, a group of strictly lytic phages is formed by myoviruses related to *Salmonella* phage Felix O1 (FO1) with genomes of ca. 85-90 kb in size. They are classified as members of the subfamily *Ounavirinae* [12]. The FO1-like *Salmonella* phages Mushroom and FO1a are components of commercially available phage cocktails (IntestiPhage™ and PhageGuard S™, respectively) [13, 14]. GJ1-like phages are also virulent myoviruses. ΦEcoM-GJ1 infects enterotoxigenic *E. coli* O149:H10:F4 strains [15]. Other phages related to GJ1, such as the *E. amylovora* phage vB_EamM-Y2 or *Pectobacterium carotovorum* phage PM1 have similar genome sizes of ca. 55 kb as well as broad host ranges and have been shown to effectively limit the growth of their target bacteria *in vitro* as well as *in vivo* [16, 17]. The strictly lytic N4-like phages belong to the family *Podoviridae* and have genomes of approximately 70-75 kb in size [18]. The N4-like *Escherichia* phage EC1-UPM reduces the severity of colibacillosis in chickens caused by *E. coli* O78:K80 strains [19]. SP6-like phages are virulent podoviruses of one genus (*Sp6virus*) with genomes of ca. 45 kb in size [20]. The SP6-like *Salmonella* phage UAB_Phi78, which displays a broad host range on strains of *Salmonella enterica* subsp. *enterica* serovars Typhimurium and Enteritidis, was part of a cocktail that greatly reduced *Salmonella* contamination on various food surfaces [21]. The relatively small and strictly lytic T7-like viruses (ca. 40 kb) also belong to one genus (*T7virus*) in the family *Podoviridae*. At their tail spikes, many T7-like phages have enzymes that hydrolyze bacterial exopolysaccharides, which assist in infection of the host [22–24]. The safety of the virulent T4-like myoviruses from the subfamily *Tevenvirinae* was demonstrated by oral application of phage preparations to healthy children and adults. No adverse effects were reported [25, 26]. A prominent member of the T4-like phages is *Salmonella* phage vB_SenM-S16, which possesses an extraordinarily broad host range [27]. Finally, the virulent Vi1-like phages share a myovirus morphology, with genomes of ca. 155-160 kb in size, and they belong to the new family *Ackermannviridae* [28, 29]. The Vi1-like phage LIMEstone1 has been shown to significantly reduce the incidence and severity of soft rot on potato tubers caused by *Dickeya solani* [30]. Likewise, contamination with *E. coli* O157:H7 on various foods has been shown to be reduced by EcoShield™, a commercial product composed of three phages, one of which (ECML-4) is a member of the genus *Vi1virus* [31, 32]. However, some Vi1-like phages have been reported to be generalized transducers [33] – a characteristic that is incompatible with its use in phage biocontrol and needs to be clarified beforehand.

The established groups of FO1-, GJ1-, N4-, SP6-, T4-, T7-, and Vi1-like phages all comprise strictly lytic phages, and representatives of each group have been successfully applied to control pathogenic bacteria. An early assignment of a new phage to one of these groups could therefore enable preselection to accelerate phage isolation and characterization. Genome sequencing allows identification of the phylogenetic relationships of a new isolate, but it is still associated with high costs and complex data analysis. In order to circumvent the need for complete sequencing, molecular markers (signature genes) could be studied instead. These markers should be universally encoded by all tailed phages, and, more importantly, the phylogenetic groups formed by comparisons of whole genome sequences and the corresponding markers need to be identical. There are several candidates for such signature genes, and their usefulness as molecular markers has already been demonstrated. For example, the grouping of Vi1-like phages has been corroborated by phylogenetic analysis of single signature genes (major capsid protein [MCP], DNA polymerase, DNA ligase, terminase large subunit) [29]. A phylogenetic tree of the former subfamily “*Felixounavirinae*” constructed by analysis of concatenated protein sequences (MCP, tail sheath protein, portal protein) also confirmed clustering based on whole-genome comparisons [34]. Signature genes have also been used to study viral diversity in environmental samples. For example, the diversity of T7-like cyanoviruses has been analyzed with the help of three signature genes (DNA polymerase, MCP, photosynthesis gene *psbA*), and these viruses have been specifically isolated using degenerate primers targeting the DNA polymerase gene [35]. In a comprehensive study of phages infecting enterobacteria, Grose and Casjens [11] showed that the chance of assigning a new isolate to the correct cluster by its MCP sequence is as high as 96.4%. Hence, a new phage isolate should be correctly grouped after MCP sequence analysis. Similar correlation values of 97.6% and 98.8% have been reported for the tail tape measure protein (TMP) [11, 36]. However, TMP cannot be used to classify members of the family *Podoviridae* because they do not encode the protein.

In this study, we used the major capsid protein as a molecular marker and designed degenerate primers to specifically identify members of the FO1-, GJ1-, N4-, SP6-, T4-, T7-, and Vi1-like phage groups early during the isolation process. We determined sensitivity and specificity of the newly developed MCP PCR assays and, as a proof of concept, isolated and identified phages infecting *E. coli*, *S. enterica*, and *E. amylovora*. Their presumptive grouping based on MCP PCR results was confirmed by electron microscopy (EM), pulsed-field gel electrophoresis (PFGE), and sequencing of the *mcp* gene fragment. The results demonstrate that the MCP PCR assay is a convenient and reliable tool for the quick identification of selected phages.

## Materials and methods

### Primer design

MCP amino acid sequences of the “type phages” T4, T7, N4, ΦEcoM-GJ1 (GJ1), SP6, Felix O1 (FO1), and ViI, and of a diverse set of closely related phages (Table 1) were used for the design of degenerate MCP primers (Table 2) applying the Codehop strategy [37]. Primer pairs with the lowest degeneracy scores possible were selected. All of the phages used for the design of the T4-MCP primers belonged to the T4 supercluster infecting enterobacteria presented by Grose and Casjens [11], except for phage 133, which is specific for *Acinetobacter*. They are all members of different genera of the subfamily *Tevenvirinae*. The T7-MCP primers were based on MCP sequences of a few phages infecting various species of the gammaproteobacteria, all of which are members of the T7-like cluster of the T7-supercluster. Likewise, SP6-like phages were selected from the SP6-cluster of the T7-supercluster [11]. Oligonucleotides for the specific detection of N4-like phages were designed based on MCP sequences of N4-like phages as published by Chan et al. [38]. Sequences of phages infecting bacteria other than gammaproteobacteria had to be excluded due to insufficient sequence similarity. The GJ1-specific primers were designed based on MCP sequences of members of the GJ1-like cluster [11] extended by *Shewanella* phage Spp001 [39] and *Aeromonas* phage pAh6-C [40]. The FO1 primer pair was designed based on the MCP sequences of the members of the Felix O1 cluster published by Grose and Casjens [11], except for FO1a, whose MCP sequence is identical to that of Felix O1. All of them belong to the subfamily *Ounavirinae* [12]. Finally, Vi1-like phages were selected based on their membership in the former genus “*Viunalikevirus*” suggested by Adriaenssens et al. [29].

**Table 1:** Phages used for primer design

Phage (host, MCP accession number)	
*T4-MCP*	
T4 (*Escherichia*, NP_049787)	vB_CsaM_GAP161 (*Cronobacter*, YP_006986489)
RB49 (*Escherichia*, NP_891732)	44RR2.8t (*Aeromonas*, NP_932516)
S16 (*Salmonella*, YP_007501205)	133 (*Acinetobacter*, YP_004300759)
*T7-MCP*	
T7 (*Escherichia*, NP_041997)	Kvp1 (*Kluyvera*, YP_002308412)
K1F (*Escherichia*, YP_338120)	VP3 (*Vibrio*, AFH14436)
ΦSG-JL2 (*Salmonella*, YP_001949782)	IME15 (*Stenotrophomonas*, YP_006990233)
vB_EamP-L1 (*Erwinia*, YP_007005458)	
*N4-MCP*	
N4 (*Escherichia*, YP_950534)	JA-1 (*Vibrio*, YP_008126822)
EC1-UPM (*Escherichia*, AGC31571)	VCO139 (*Vibrio*, AGI61887)
IME11 (*Enterobacter*, YP_006990615)	VBP32 (*Vibrio*, YP_007676568)
EcP1 (*Enterobacter*, YP_007003179)	Presley (*Acinetobacter*, YP_009007653)
FSL_SP-076 (*Salmonella*, YP_008240197)	pYD6-A (*Pseudoalteromonas*, YP_007674292)
FSL_SP-058 (*Salmonella*, YP_008239469)	LUZ7 (*Pseudomonas*, YP_003358361)
vB_EamP-S6 (*Erwinia*, YP_007005821)	LIT1 (*Pseudomonas*, YP_003358474)
VBP47 (*Vibrio*, YP_007674146)	PA26 (*Pseudomonas*, AFO70574)
*GJ1-MCP*	
ϕEcoM-GJ1 (*Escherichia*, YP_001595448)	vB_EamM-Y2 (*Erwinia*, YP_007004718)
PM1 (*Pectobacterium*, YP_009021819)	Spp001 (*Shewanella*, YP_009008839)
pAh6-C (*Aeromonas*, YP_009103334)	
*SP6-MCP*	
SP6 (*Salmonella*, NP_853592)	K1-5 (*Escherichia*, YP_654132)
UAB_Phi78 (*Salmonella*, YP_007501015)	PP1 (*Pectobacterium*, YP_007010676)
K1E (*Escherichia*, YP_425009)	Era103 (*Erwinia*, YP_001039668)
vB_EcoP-ACG-C91 (*Escherichia*, YP_006987798)	
*FO1-MCP*	
Felix O1 (*Salmonella*, NP_944891)	JH2 (*Escherichia*, YP_009219540)
UAB_Phi87 (*Salmonella*, YP_009150189)	ΦEa104 (*Erwinia*, YP_004327026)
EC6 (*Escherichia*, YP_009151278)	ΦEa21-4 (*Erwinia*, YP_002456075)
wV8 (*Escherichia*, YP_002922847)	vB_EaM-M7 (*Erwinia*, AEJ81281)
*Vi1-MCP*	
ViI (*Salmonella*, YP_004327523)	PhaxI (*Escherichia*, YP_007002787)
SFP10 (*Salmonella*, YP_004895313)	ΦSboM-AG3 (*Shigella*, YP_003358645)
ΦSH19 (*Salmonella*, YP_007008101)	vB_DsoM_LIMEstone1 (*Dickeya*, YP_007237460)
CBA120 (*Escherichia*, YP_004957847)

**Table 2:** Primers designed and used in this study

Name	Sequence (5’→3’)	Degeneracy score
T4-fw	CCC TGC TGT TCC AGA TCG ANA ARG ARG C	16
T4-rev	CTG CCT GGC GTA CTG GTC DAT RWA NAC	48
T7-fw	GAC AAG CGG AAG GAC ATC AAN CAY ACN GAR A	64
T7-rev	CGC GTA GTT GGC GGC RTT NGG CAT NA	32
N4-fw	GGA TGA TCG TAA TAT TAA TGA TCA GGG NAT HRA YGC	48
N4-rev	GAC ATA AAG CCC ATT TCG CCR WAN GGR TC	32
GJ1-fw	GGC TGC GCG TAT GAT TAG GAY ATH GAY GA	12
GJ1-rev	CCA ATG CAT CAC CGG CAD CCA DAT YTC	18
SP6-fw	CAC CGT GAT TGC GCG TAA YAC NGT NGC	32
SP6-rev	TTC CCA ACG ATC CGG AAT NGC NCC YTC	32
FO1-fw	CGC CAT TGA AGA ACT GCG TRW RCA YAT GGA	16
FO1-rev	GGC ATC ATA TAG GAA TGC GCY TCR AAR TC	8
Vi1-fw	GCC GAT TAA TAT TGC GAT GGA YTT YTT	4
Vi1-rev	CCA GCA TAA AGG TCA TAA ATT TCC AYT TYT C	4

### Dotplot and phylogenetic analysis

Dotplot analysis of MCP sequences used for primer design was performed with Gepard using standard parameters [41]. A phylogenetic tree was constructed with CLC Main Workbench (version 7.9.1, QIAGEN Bioinformatics, Aarhus, Denmark), applying the neighbor-joining algorithm and bootstrap analysis. Partial *mcp* gene sequences of the new isolates were included to study their phylogenetic relationships.

### Preparation of templates (plaque PCR)

Phages were cultivated using the soft-agar overlay method [8] with temperatures and media listed in Table S1. Well- separated single plaques were picked with a Pasteur pipette, and the agar plugs were transferred to PCR test tubes containing 100 µl of SM buffer (50 mM Tris, 100 mM NaCl, 8 mM MgSO_4_, pH 7.4). The tubes were incubated for 1 h at room temperature to allow diffusion of the phages into the buffer. Aliquots of 50 µl were removed for any subsequent analysis requiring intact virus particles, and the remaining 50 µl was heated in a thermocycler for 10 min at 95 °C and cooled down to 8 °C. This caused melting of the agar plug and release of the phage particles into the buffer. Two microliters of the heat-treated samples was used as template for the PCR assay.

### PCR conditions

Phages T4, T7, and N4 were used as positive controls for the respective PCRs. The positive controls of the other PCRs were *Salmonella* phage FO1-E2 (FO1-specific PCR; Marti et al., unpublished), *Erwinia* phage vB_EamP-S2 (SP6; [17, 42]), *Erwinia* phage vB_EamM-Y2 (GJ1; [17]), and *Salmonella* phage KCK6 (Vi1; Born et al., unpublished). KAPA Taq ReadyMix (Sigma-Aldrich, Buchs, Switzerland) was used for PCR following the manufacturer’s instructions. Reactions were performed in 20-µl volumes (10 µl of KAPA Taq, 0.8 µl of each primer [10 µM], 6.4 µl of ddH_2_O, and 2 µl of template) with an annealing temperature of 50 °C (T4, FO1, GJ1, Vi1) or 55 °C (N4, SP6, T7).

### Detection limits

For determination of the detection limits, plaques of the respective positive controls were picked and resuspended in 100 µl of SM buffer as described above. After 1 h, phages were serially diluted, and the concentrations of infectious virions were determined using soft-agar overlays. Immediately thereafter, the same dilution series was heated to 95 °C for 10 min and used as a template for PCR. These experiments were independently performed twice.

### Multiplex PCR

Reactions generated positive results with annealing temperatures over a wide range. To increase the throughput of the method, screening was performed in multiplex PCRs. MCP PCR format with similar annealing temperatures but generating fragments of different sizes that allowed clear visual discrimination after gel electrophoresis were combined. Conditions were established to run triplex PCRs in 20-µl volumes (10 µl of KAPA Taq, 0.8 µl of each primer (10 µM), 3.2 µl of ddH_2_O, and 2 µl of template). T4/T7/SP6 (annealing temperature: 52 °C) and N4/FO1/GJ1 (54 °C) were successfully combined, whereas SP6 could also be replaced by Vi1. Detection limits were not determined, but picking single plaques and running the PCRs according to the standard protocol was sufficient to generate positive results.

### Specificity/sensitivity testing

A set of 26 phages (Table 3) were tested to evaluate the sensitivity and specificity of the method. Phages were cultivated (Table S1) and picked as described above. PCRs were performed in single reactions and repeated once.

**Table 3:** Complete list of phages used for performance testing

Name	Family^a^	Subfamily	Genus	Host	Lifestyle^b^	Genome size (kb)	Reference
vB_EamP-L1	*P*	*Autographivirinae*	*T7virus*	*Erwinia*	v	39.3	[17]
T7	*P*	*Autographivirinae*	*T7virus*	*Escherichia*	v	39.9	DSMZ^c^
vB_EamP-S2	*P*	*Autographivirinae*	*SP6virus*	*Erwinia*	v	45.5	[17, 42]
N4	*P*	*-*	*N4virus*	*Escherichia*	v	70.2	[18]
vB_EamP-S6	*P*	*-*	-	*Erwinia*	v	74.7	[17]
φ29	*P*	*Picovirinae*	*Phi29virus*	*Bacillus*	v	19.3	[63]
P22	*P*	*-*	*P22virus*	*Salmonella*	t	41.7	DSMZ
vB_EamM-Y2	*M*	-	-	*Erwinia*	v	56.6	[17]
vB_EamM-M7	*M*	*Ounavirinae*	*Ea214virus*	*Erwinia*	v	84.7	[17]
FO1-E2	*M*	*Ounavirinae*	*Felixo1virus*	*Salmonella*	v	83.3	Marti et al.^d^
JG004	*M*	*-*	*Pakpunavirus*	*Pseudomonas*	v	93.0	[64]
S16	*M*	*Tevenvirinae*	*S16virus*	*Salmonella*	v	160.2	[27]
T4	*M*	*Tevenvirinae*	*T4virus*	*Escherichia*	v	168.9	DSMZ
LBL3	*M*	*-*	*Pbunavirus*	*Pseudomonas*	v	64.4	[65]
A511	*M*	*Spounavirinae*	*P100virus*	*Listeria*	v	137.6	[44, 66]
P100	*M*	*Spounavirinae*	*P100virus*	*Listeria*	v	131.4	[66, 67]
K	*M*	*Spounavirinae*	*Kayvirus*	*Staphylococcus*	v	148.3	[66, 68, 69]
P2	*M*	*Peduovirinae*	*P2virus*	*Escherichia*	t	33.6	[70]
TK611	*S*	-	*T5virus*	*Salmonella*	v	120.9	Born et al.^d^
λ	*S*	-	*Lambdavirus*	*Escherichia*	t	48.5	DSMZ
P35	*S*	*-*	-	*Listeria*	v	35.8	[71]
P40	*S*	*-*	-	*Listeria*	v	35.6	[71]
P70	*S*	-	*P70virus*	*Listeria*	v	67.2	[72]
A500	*S*	-	-	*Listeria*	t	38.9	[72]
A118	*S*	-	-	*Listeria*	t	40.8	[73]
KCK6	*A*	*Cvivirinae*	*Vi1virus*	*Salmonella*	v	158.5	Born et al.^d^

^a^*P*, *Podoviridae*; *M*, *Myoviridae*; *S*, *Siphoviridae; A, Ackermannviridae*

^b^v, virulent (strictly lytic); t, temperate

^c^DSMZ, Deutsche Sammlung von Mikroorganismen und Zellkulturen (German Collection of Microorganisms and Cell Culture)

^d^unpublished data

### Phage isolation

Phages were isolated from various environmental samples (ponds, sewage water, soil, and animal feces). Solid samples were mixed with 10 ml of SM buffer per gram and homogenized. Solid particles were removed by centrifugation. Enrichment was performed in volumes of 20 ml and contained 10 ml of 2 × concentrated LB broth, 2 ml of SM buffer (10 ×), 2 ml of a bacterial overnight culture, and 6 ml of the preprocessed environmental sample. After overnight incubation at the desired temperature, bacteria and other particles were removed by centrifugation (2 min, 10,000 × *g*), and the supernatants were sterilized using 0.2-µm-pore-size filters. To obtain single plaques, dilution series were plated using the soft-agar overlay method [8]. Incubation temperatures and media used to propagate the new phage isolates are listed in Table S1. Single plaques were then analyzed by multiplex MCP PCR. Positive results were confirmed by PCR using the corresponding primer pair only.

### Phage purification and characterization

MCP-PCR-positive phages were propagated and purified using CsCl density gradient centrifugation as described elsewhere [17, 43]. Electron microscopy of negatively stained phage particles was performed as described earlier [44]. For the extraction of phage DNA, CsCl-purified virus particles were dialyzed against a 1,000-fold excess of SM buffer (6 h, RT). Free nucleic acids were degraded by treatment for 15 min at 37 °C with DNase I (final concentration: 1 U/ml) and RNase A (50 µg/ml). Nucleases were inactivated and phage capsids were degraded by treatment for 1 h at 56 °C with EDTA (20 mM, pH 8.0), proteinase K (50 μg/ml), and SDS (0.5% [wt/vol]), and DNA was purified using a phenol-chloroform extraction procedure with subsequent ethanol precipitation [43]. Genome sizes were determined by PFGE (settings: 6 V/cm, switch times: 2-25 s, 20 h, 14 °C) using a Chef DR III apparatus (Bio-Rad, Reinach, Switzerland).

## Results

### Distinct clusters revealed by MCP sequence comparisons

**Fig. 1:**
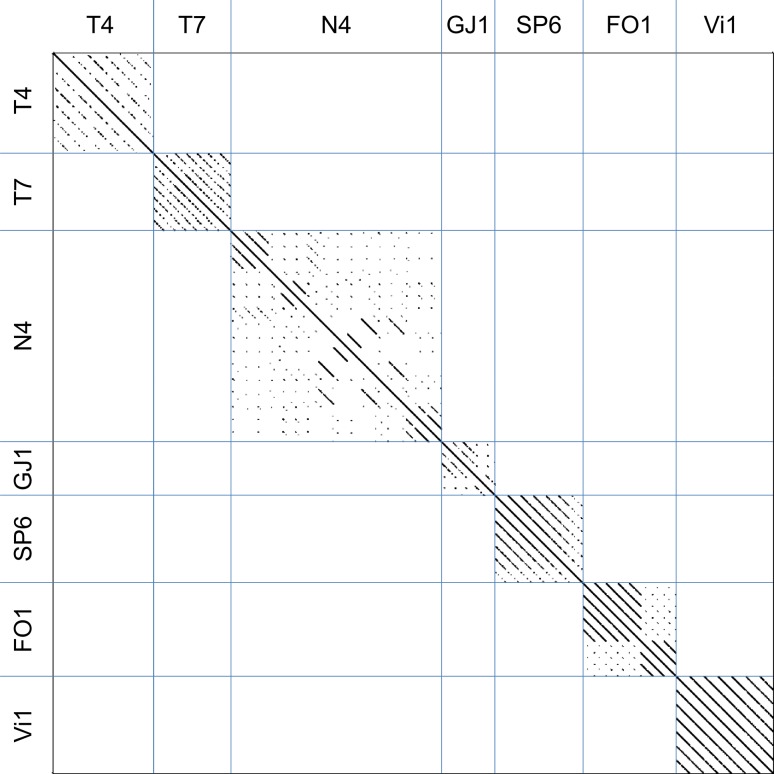
Dotplot analysis of MCP sequences used for the primer design. The order of the phages is according to Table 1

**Fig. 2:**
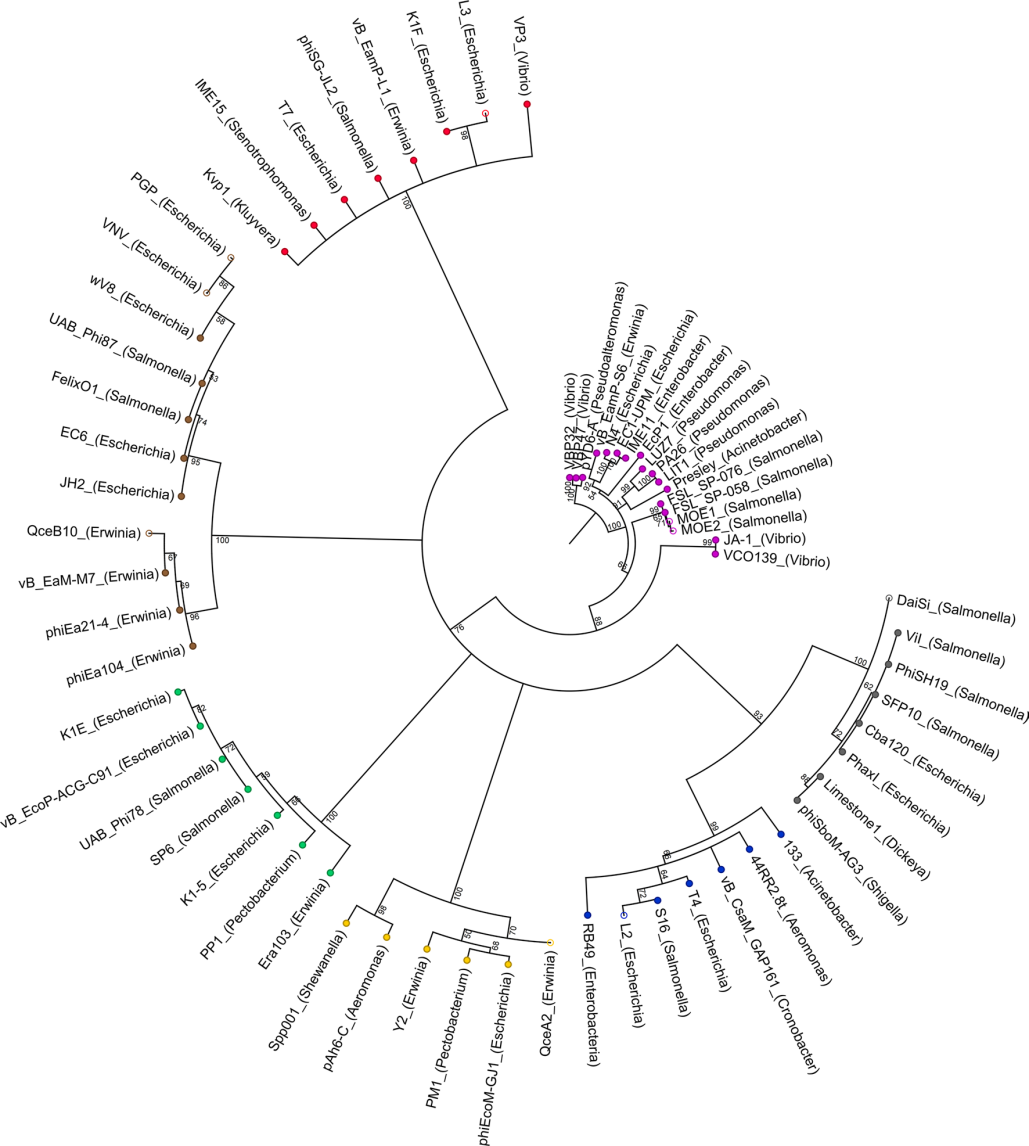
Phylogenetic analysis of novel phages. New MCP sequences were aligned with those used for primer design. A neighbor-joining tree was constructed with 1,000 bootstrap replicates. Branches with values < 50% were collapsed. Nodes are colored according to phage groups. Purple, N4; grey, Vi1; blue, T4; yellow, GJ1; green, SP6; brown, FO1; red, T7. Open nodes indicate novel phages

The MCP sequences that were used for the primer design were compared by dotplot analysis (Fig. 1). The seven phage types studied formed distinct clusters with clear similarities only within each group. Subclusters were observed in the groups of the N4-, GJ1-, and FO1-like phages. There were two subclusters in the GJ1 and FO1 groups, which correlated well with the host specificities of the corresponding phages. Phages infecting enterobacteria were separate from those infecting non-enterobacteria and such specific for *Escherichia* and *Salmonella* were separate from the *Erwinia* phages. Similar tendencies were observed for the N4-like phages. However, the subclusters were not as distinct. These clear clusters were also prominent in the phylogenetic tree (Fig. 2).

### Direct analysis of plaques by MCP PCR

The detection limit was defined as the lowest number of PFUs needed to produce a visible band in gel electrophoresis after PCR. The limits ranged from 10^1^ to 10^3^ PFU per reaction, which is equivalent to using a template concentration of 10^4^ to 10^6^ PFU/ml. Since we used a tenfold dilution series without further gradation, the actual limits might have been slightly lower. Detection limits were not determined for the multiplex assays. Most importantly, the phage DNA concentrations in the picked and heated agar plugs were sufficient in all cases to generate positive PCR results. In the analysis of the 26 well-characterized phages, not a single false negative or false positive result was obtained (data not shown). Thus, both the specificity and the sensitivity of all primer pairs were 100%. A few reactions resulted in nonspecific results, seen as faint bands that differed in size from the positive controls. We rated these as negative.

### Low environmental frequency of the targeted phage groups revealed by multiplex MCP PCR

*Salmonella*, *Escherichia*, and *Erwinia* phages were isolated from different environmental samples. In total, 154 plaques were picked and analyzed. Of these, 14 belonged to one of the seven groups of interest based on MCP PCR analysis (Table 4). More precisely, *Escherichia* phage L2 was a presumptive T4-like isolate, while the T7-MCP PCR was positive when using phage L3 as a template. *Salmonella* phages MOE1 and MOE2 were potential N4-like phages. *Erwinia* phage QceA2 was found to be GJ1-positive. *Escherichia* phages PGP and VNV were presumptive FO1-like phages, as were the other six positive *Erwinia* samples. Since the *Erwinia* samples were collected from the same quince orchard, they were likely to be identical. Thus, only QceB10 was selected, and we did not analyze the remaining FO1-positive *Erwinia* isolates. *Salmonella* phage DaiSi was a presumptive Vi1-like phage. The initial grouping of the new phage isolates was confirmed by estimating their genome sizes using PFGE and analysis of their morphologies by EM (except for L3, which we failed to propagate to high titers). The results are summarized in Table 4, while the original data from PFGE analysis (Fig. S1) and electron micrographs (Fig. S2) are provided as supplementary material. In addition, PCR products of the nine selected phages were sequenced and compared by blastx [45] with the non-redundant GenBank database. The closest relative of the presumptive T4-like *Escherichia* phage L2 was found to be *Escherichia* phage vB_EcoM_VR26 (100% amino acid sequence identity) [46], which belongs to the genus *Sp18virus* of the subfamily *Tevenvirinae*. L3 was most closely related to *Escherichia* phage LM33_P1 (100%), a member of the genus *T7virus* [47]. The MCP sequences of the putative N4-like *Salmonella* phages MOE1 and MOE2 shared 99% amino acid sequence identity with *Salmonella* phage FSL SP-058. FSL SP-058 was proposed to be grouped into the genus *Sp58virus* of N4-like viruses [18, 48]. *Erwinia* phage QceA2, which was isolated as a presumptive GJ1-like phage, shared 99% amino acid sequence identity with *Erwinia* phage vB_EamM-Y2, which is closely related to phage ΦEcoM-GJ1 [11, 17]. The presumptive FO1-like isolates PGP and VNV were found to be most similar to *Salmonella* phage UAB_Phi87 (99%) and *Escherichia* phage vB_EcoM-VpaE1 (100%), respectively. Both are members of the genus *Felixo1virus* in the subfamily *Ounavirinae* [12, 49, 50]. *Erwinia* phage vB_EamM-M7, the closest relative of the putative FO1-like *Erwinia* phage QceB10 (99%), belongs to the same subfamily but to another genus (*Ea214virus*) [12]. The presumptive Vi1-like *Salmonella* phage DaiSi shared a 99% amino acid sequence identity with *Salmonella* phage FSL SP-029, which is also related to ViI [48]. Phylogenetic analysis of the partial MCP sequences also confirmed classification of the novel phages into the expected groups (Fig. 2).

**Table 4:** Properties of isolated phages. Presumptive grouping is based on MCP PCR analysis. Genome sizes were determined by PFGE (Fig. S1); morphology was analyzed by electron microscopy (Fig. S2). nd: not determined

Isolate	Host	Presumptive grouping	Expected		Experimental confirmation	
			Genome size	Family^a^	Genome size	Tail morphology
L2	*E. coli*	T4	160-175 kb	*M*	160 kb	contractile
L3	*E. coli*	T7	40 kb	*P*	nd	nd
MOE1	*S.* Typhimurium	N4	70-75 kb	*P*	72 kb	short
MOE2	*S.* Typhimurium	N4	70-75 kb	*P*	72 kb	short
QceA2	*E. amylovora*	GJ1	55 kb	*M*	55 kb	contractile
PGP	*E. coli*	FO1	85-90 kb	*M*	85 kb	contractile
VNV	*E. coli*	FO1	85-90 kb	*M*	85 kb	contractile
QceB10	*E. amylovora*	FO1	85-90 kb	*M*	83 kb	contractile
DaiSi	*S.* Typhimurium	Vi1	155-160 kb	*A*	158 kb	contractile

^a^*P*, *Podoviridae*; *M*,*Myoviridae*; *A, Ackermannviridae*

The partial MCP sequences of the isolated phages were deposited in the GenBank database under the accession numbers MK163343 (L2), MK163344 (L3), MK163345 (MOE1), MK163346 (MOE2), MK163347 (QceA2), MK163348 (PGP), MK163349 (VNV), MK163350 (QceB10), and MK163351 (DaiSi).

## Discussion

Due to the enormous diversity of bacteriophages in nature, the search for new phages with high potential for biocontrol purposes is rather limited. A new phage isolate needs to fulfill important requirements, such as being strictly lytic, specific, safe, and non-transducing, if applied in biocontrol. In addition, as demonstrated here, the FO1-, GJ1-, N4-, SP6-, T4-, T7-, and Vi1-like phages seem to be present at only a low frequency in environmental samples. A comprehensive analysis of phages infecting enterobacteria showed that members of the same cluster share the same lifestyle [11]. A simple classification of new isolates similar to the 16S rRNA analysis of bacteria could thus facilitate the identification of new candidates. The approach could be hampered by the mosaic architecture of phage genomes due to extensive horizontal gene transfer [5], which could lead to random isolation if classification relied on a single gene only. However, horizontal transfer of certain genes such as those encoding the head proteins is limited, because they interact in building essential structures. Random recombination events can lead to non-functional hybrids that are then lost from the population [51]. Consequently, genes encoding proteins such as the major capsid protein or the tail tape measure protein are conserved and can be used as signature genes for classification of phages [11, 36]. In this study, we selected MCP as a molecular marker for early assignment of new phages to a certain group and thus for a preliminary identification of their lifestyle. There is a high correlation (96.4%) between MCP types and whole-genome clusters of enterobacteria phages, as shown by Grose and Casjens [11]. Eleven of the twelve exceptions resided in the lambda supercluster. These exceptions are irrelevant for our primers, which were designed based on only strictly lytic phages. Also, there is no cluster that contains both strictly lytic and temperate phages, and no phage has recently switched between the two lifestyles [11].

None of the 26 phages with known classification gave false positive results in our performance testing. This collection was greatly expanded by our screening of environmental isolates. In every case, the closest relatives deduced from sequence comparisons were members of the expected groups. Genome sizes determined by PFGE further supported the initial identification, and EM-based analysis of the morphology of each isolate was in agreement with the MCP PCR prediction. We consider these analyses sufficient to confirm the presumptive MCP PCR results, since both genome sizes and tail types within clusters are uniform [11]. Thus, there were no false positive results, i.e., the specificity was 100%. This finding further indicates that the degeneracy of the primers did not cause nonspecific annealing. False negatives, however, would not have been identified in the field study owing to the experimental setup. In theory, false negative results could originate from insufficient sequence similarity for primer binding, impurities, or concentrations below the detection limit. Lack of sensitivity is most probably not an issue for the new method presented here. Picking a plaque using a Pasteur pipette and resuspending it in 1 ml of SM buffer usually yields a final concentration of > 10^6^ PFU/ml. The detection limits ranged from 10^1^-10^3^ PFU per reaction (equal to a required template concentration of 10^4^-10^6^ PFU/ml), which is in the same range as the detection limits reported in other studies [34, 35]. In fact, all positive control PCRs performed with an undiluted template were clearly positive, demonstrating that picking a plaque and using it according to the presented protocol does not produce false negative results in either single or multiplex reactions.

All primers used in this study were designed based on sequences of virulent phages (Table 1). Depending on the current taxonomy of these phages (which is highly dynamic in some cases [52]), the specificity of each primer set is at a certain taxonomic level. Alignment of the MCP sequences of all members of the T4 cluster indicated conservation of certain regions [11]. The T4 MCP primers target such regions. Their design is based on a few phages of different genera of the subfamily *Tevenvirinae*, which comprises a constantly growing number of genera whose members are specific for gammaproteobacteria [10, 53]. The T4 MCP PCR assay is therefore expected to identify phages of the subfamily *Tevenvirinae* that infect gammaproteobacteria. *Escherichia* phage vB_EcoM_VR26, the closest relative of the new isolate L2, belongs to the genus *Sp18virus* of the subfamily *Tevenvirinae* [46].

T7 and SP6 are the prototype phages of their respective genera (*T7virus* and *SP6virus*). Both belong to the subfamily *Autographivirinae* [20]. The T7- and SP6-MCP PCRs are intended to detect phages at the genus level. The T7-MCP PCR could not amplify the SP6-prototype virus and vice versa, confirming the genus-specificity of these two MCP PCRs. *Escherichia* phage LM33_P1, which was found to be the closest relative of L3, is a member of the genus *T7virus* [47]. A SP6-like phage could not be isolated in our study.

It is difficult to clearly pinpoint the specificity to a certain taxon in the case of the N4-MCP PCR. The phages used for the design of our N4 primers belong to three genera of N4-like phages (*G7cvirus*, *Lit1virus*, *Sp58virus*), whereas only *G7cvirus* belongs to the proposed subfamily “*Enquartavirinae*” [18]. The fourth proposed genus of N4-like phages, “*Dss3virus*”, consisting of two viruses infecting *Ruegeria* spp. and *Sulfitobacter* spp., respectively, was not represented in our selection due to insufficient sequence similarity. Phage FSL SP-058, which is related to the isolated phages MOE1 and MOE2, is a member of the genus *Sp58virus* [48]. Wittmann et al. argued that the N4-like phages belong to a higher taxonomic division than a genus or subfamily [18]. This is also strengthened by dotplot analysis of the N4-like MCP sequences, which resulted in more subgroups than did comparisons of the other MCP-sequences. Thus, our N4 MCP PCR amplifies viruses of more than one genus and is specific for the group formerly known as “N4-like phages” infecting gammaproteobacteria [38].

The genus “*GJ1virus*” has not yet been established. The five phages used for the design of the GJ1 MCP primers share ≥ 40% protein homologs with ΦEcoM-GJ1 as calculated by CoreGenes 3.5 [54], i.e., they fulfill an important criterion that was used in the past to group phages into the same genus [10]. However, the number of homologous proteins using ΦEcoM-GJ1 as reference varies considerably (PM1, 81%; Y2, 59%; Spp001, 48%; pAh6-C, 40%), and dotplot analysis of MCP sequences revealed subclusters. Thus, more sequences of GJ1-like phages are required to clarify the taxonomy of the GJ1-like phages and thereby the specificity of this PCR assay. *Erwinia* phage QceA2, which was isolated as a presumptive GJ1-like phage, shared the highest amino acid sequence similarity with the MCP of *Erwinia* phage vB_EamM-Y2, which is closely related to phage ΦEcoM-GJ1 [11, 17].

The group of FO1-like phages was reassorted in the past. All phages used for primer design are members of the subfamily *Ounavirinae*, which was recently established [12]. Related phages of the genera *Pakpunavirus* and KPP10virus and the proposed genus “*KILvirus*” infect *Pseudomonas* strains only [34, 55]. A BLAST-search of the FO1-primers against the MCP sequences of the PAK_P1-, KPP10-, and KIL-like clades was negative. We thus expect that the FO1-primers only amplify MCP genes of FO1 like phages infecting enterobacteria. Hence, the FO1 MCP PCR specifically identifies members of the subfamily *Ounavirinae*. This was further supported by partial MCP sequences of the presumptive FO1-like isolates PGP, VNV, and QceB10. PGP and VNV were found to be most similar to *Salmonella* phage UAB_Phi87, and to *Escherichia* phage vB_EcoM-VpaE1, respectively, both of which are members of the genus *Felixo1virus* [12, 49, 50]. *Erwinia* phage vB_EamM-M7, the closest relative of QceB10, belongs to the genus *Ea214virus* [12].

Finally, the Vi1 MCP primers were originally designed based on phages of a single genus [29]. A recent reclassification has placed them into two separate subfamilies of a novel family [56]. Thus, the Vi1 MCP PCR selects members of the new family *Ackermannviridae*. The isolate DaiSi was found to be related to *Salmonella* phage FSL SP-029, a Vi1-like phage [48].

MCP-PCR-based detection of phages can most probably be adapted to any phage genus or subfamily of interest, provided that sufficient sequence data are available. Consequently, the MCP PCR assay is not suited to identify phages of uncharacterized groups. Despite its rather limited detection range, the MCP PCR assay still allows the discovery of novel phages, because two phages of the same genus do not necessarily share all properties, e.g., host ranges within the target species can differ considerably if two isolates exhibit different tail structures. For example, there is a high variability in genes showing similarities to tail spikes in the N4-like phages, which might be responsible for differences in host specificity [18]. Likewise, the genomes of many podoviruses infecting members of the genus *Acinetobacter* exhibit high homology and collinearity except for a pectate lyase domain located in their tail fibers, which is responsible for differences in host range [57]. Vi1-like phages also exhibit significant divergence only in their tail spike regions [29]. Depending on their tail structures, Vi1-like phages with high specificity for certain serotypes as well as those infecting members of different genera have been described [58–62]. Finally, the T4-like *Salmonella* phage S16 which has an extraordinarily broad host range, is characterized by long tail fibers with an unexpected architecture known from T2-like phages [27].

Considering the enormous diversity of bacteriophages in nature, it is very challenging to specifically isolate a phage that perfectly meets all requirements for biocontrol purposes. Thus, an MCP-PCR-based preselection at the very beginning of the isolation process clearly facilitates the search for novel biocontrol phages. Once selected for further applications, the complete phage genome needs to be sequenced to confirm the preliminary classification and to ultimately prove the strictly lytic lifestyle. This also enables identification of potential toxin genes. In addition, the transduction potential of the phage needs to be addressed. Altogether, we conclude that the MCP PCR is a useful method for targeted isolation of selected virulent phages and sorting out of undesired isolates, such as frequently found temperate phages.

## Electronic supplementary material

Below is the link to the electronic supplementary material.

Supplementary material 1 (DOCX 1349 kb)
