# Supplementary material for: A major-capsid-protein-based multiplex PCR assay for rapid identification of selected virulent bacteriophage types
Source: Arch Virol. 2019 Jan 23;164(3):819–30. doi: 10.1007/s00705-019-04148-6 (PMC6394723; doi:10.1007/s00705-019-04148-6)

Table S1 Cultivation conditions of phages used in this study

| **Phage** | **Host strain** | **Medium*** | | **Temperature (°C)** | |
| --- | --- | --- | --- | --- | --- |
|  |  | **Bottom agar** | **Soft agar** |  |  |
| *Isolates from internal phage collection* | | | | | |
| vB_EamP-L1 | *E. amylovora* CFBP 1430 | LB | LC | | 28 |
| vB_EamP-S2 | *E. amylovora* CFBP 1430 | LB | LC | | 28 |
| vB_EamP-S6 | *E. amylovora* 4/82 | LB | LB+ | | 28 |
| vB_EamM-Y2 | *E. amylovora* 4/82 | LB | LB+ | | 28 |
| vB_EamM-M7 | *E. amylovora* 4/82 | LB | LB+ | | 28 |
| T7 | *E. coli* DSM 498 | LB | LB+ | | 37 |
| N4 | *E. coli* DSM 498 | LB | LB+ | | 37 |
| T4 | *E. coli* DSM 613 | LB | LB+ | | 37 |
| P2 | *E. coli* DSM 1576 | LB | LB+ | | 37 |
| λ | *E. coli* DSM 498 | LB | LB+ | | 37 |
| FO1-E2 | *S.* Typhimurium DT7155 | LB | LB+ | | 37 |
| S16 | *S.* Typhimurium DT7155 | LB | LB+ | | 37 |
| KCK6 | *S.* Typhimurium DT7155 | LB | LB+ | | 37 |
| TK611 | *S.* Typhimurium DT7155 | LB | LB+ | | 37 |
| P22 | *S.* Typhimurium ATCC 700720 | LB | LB+ | | 37 |
| LBL3 | *P. aeruginosa* PAO1 | LB | LB+ | | 37 |
| JG004 | *P. aeruginosa* PAO1 | LB | LB+ | | 37 |
| A511 | *L. ivanovii* WSLC 3009 | LC | LB+ | | 30 |
| P100 | *L. ivanovii* WSLC 3009 | LC | LB+ | | 30 |
| P40 | *L. ivanovii* WSLC 3009 | LC | LB+ | | 30 |
| P70 | *L. ivanovii* WSLC 3009 | LC | LB+ | | 30 |
| P35 | *L. monocytogenes* WSLC 1001 | LC | LB+ | | 30 |
| A500 | *L. monocytogenes* WSLC 1042 | LC | LB+ | | 30 |
| A118 | *L. monocytogenes* WSLC 1001 | LC | LB+ | | 30 |
| K | *S. aureus* DSM 799 | LB | LB+ | | 37 |
| φ29 | *B. subtilis* BD170 | LB | LB+ | | 30 |
| *New isolates* |  |  |  | |  |
| QceA2 | *E. amylovora* 4/82 | LB | LB+ | | 28 |
| QceB10 | *E. amylovora* 4/82 | LB | LB+ | | 28 |
| PGP | *E. coli* DSM 498 | LB | LB+ | | 37 |
| VNV | *E. coli* DSM 498 | LB | LB+ | | 37 |
| L2 | *E. coli* 264 (O157) | LB | LB+ | | 37 |
| L3 | *E. coli* P11-2315 (O103) | LB | LB+ | | 37 |
| MOE1 | *S.* Typhimurium DT7155 | LB | LB+ | | 37 |
| MOE2 | *S.* Typhimurium DT7155 | LB | LB+ | | 37 |
| DaiSi | *S.* Typhimurium DT7155 | LB | LB+ | | 37 |

*LB+ is LB supplemented with MgSO_4_ (2 mM) and CaCl_2_ (10 mM); LC is LB+ with 1% glucose

**Fig. S1** PFGE-analysis of phage genomes (as indicated). The DNA length ruler (first and last lane) is composed of genomes of the following phages: S16 (*Salmonella*, 160.2 kb); TK611 (*Salmonella*, 120.9 kb); FO1-E2 (*Salmonella*, 83.3 kb); N4 (*Escherichia*, 70.2 kb); λ (*Escherichia*, 48.5 kb); T7 (*Escherichia*, 39.9 kb); φ29 (*Bacillus*, 19.3)


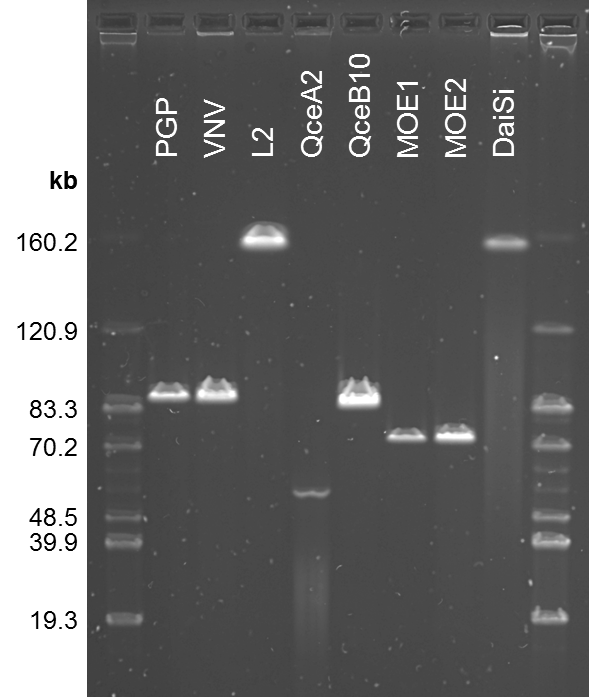


**Fig. S2** Electron micrographs of negatively stained phage particles. Top row: L2, MOE1, MOE2, A2. Bottom row: PGP, VNV, B10, DaiSi. The length ruler is 100 nm


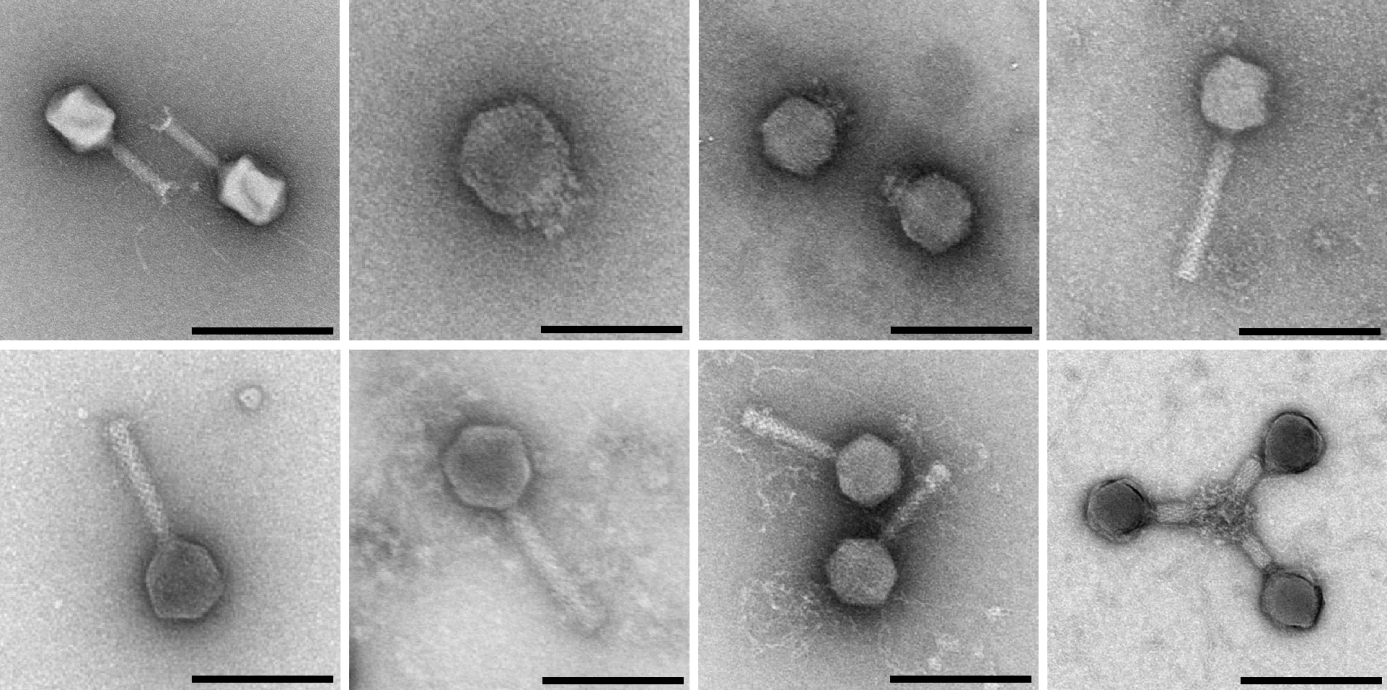

Supplement: Supplementary file 1 — Supplementary material 1 (DOCX 1349 kb) [file 705_2019_4148_MOESM1_ESM.docx]
